# Fabrication and Characterization of Multiscale PLA Structures Using Integrated Rapid Prototyping and Gas Foaming Technologies

**DOI:** 10.3390/nano8080575

**Published:** 2018-07-27

**Authors:** Byung Kyu Park, David J. Hwang, Dong Eui Kwon, Tae Jun Yoon, Youn-Woo Lee

**Affiliations:** 1 Institute of Advanced Machine and Design, Seoul National University, Seoul 151-744, Korea; 2 Department of Mechanical Engineering, State University of New York, Stony Brook, NY 11794, USA; 3 School of Chemical and Biological Engineering, Institute of Chemical Process, Seoul National University, Seoul 151-744, Korea; sot5656@snu.ac.kr (D.E.K.); marteacherth@snu.ac.kr (T.J.Y.)

**Keywords:** multiscale structure, rapid prototyping, nanofoam, gas foaming, polylactic acid

## Abstract

Multiscale structured polymers have been considered as a promising category of functional materials with unique properties. We combined rapid prototyping and gas foaming technologies to fabricate multiscale functional materials of superior mechanical and thermal insulation properties. Through scanning electron microscope based morphological characterization, formation of multiscale porous structure with nanoscale cellular pores was confirmed. Improvement in mechanical strength is attributed to rearrangement of crystals within CO_2_ saturated grid sample. It is also shown that a post-foaming temperature higher than the glass transition temperature deteriorates mechanical strength, providing process guidelines. Thermal decomposition of filament material sets the upper limit of temperature for 3D printed features, characterized by simultaneous differential scanning calorimetry and thermogravimetric analysis. Porosity of the fabricated 3D structured polylactic acid (PLA) foam is controllable by suitable tuning of foaming conditions. The fabricated multiscale 3D structures have potential for thermal insulation applications with lightweight and reasonable mechanical strength.

## 1. Introduction

Polylactic acid (PLA) is a thermoplastic aliphatic polyester polymer that is derived from renewable resources. It has received increased attention as a potential environmentally friendly substitute for petroleum-based counterparts. It is excellent for food contact, packaging, and scaffold applications, and among the polymers with the broadest ranges of applications, especially due to its ability to be stress crystallized, thermally crystallized, impact modified, filled, copolymerized, and processed in most polymer processing equipment. There has been an increasing demand for technologies to improve energy efficiency of buildings [1,2,3], and PLA has additional merits that make it unique in the marketplace, e.g., an innovative PLA bead foam technology in thermal insulation. Life-cycle assessment conducted on various thermal insulating products has also identified PLA as an interesting alternative to fossil resource-dependent resins [4]. Thermoplastic foams can serve as lightweight materials with desirable mechanical and thermal properties, and flexibility for wide range of applications [5,6,7] including thermal insulation field. It is important to have proper mechanical properties to maintain the shape when external forces are applied to such materials. The closed pore structure maintains porosity even when the material is deformed, which is a very important factor in maintaining the initial material characteristics [8,9]. While PLA based freeform is composed of flexible connecting structures that can maintain constant bearing capacity, further functionality can be added through postprocessing. Senatov et al. [10] made porous scaffold with pre-modeled PLA structure by fused filament fabrication and investigated the mechanical properties and shape memory effect. Zhou et al. [11] combined fused deposition and gas foaming technique to fabricate polymer scaffolds and characterized the microscale pore structure. Wang et al. [12] fabricated tunable nanocelluar polymethylmethacrylate (PMMA)/thermoplastic polyurethane (TPU) polymer structures and obtained a remarkably enhanced toughness and high compressive strength.

On the other hand, macroscale three-dimensional mesh structure, which can be easily achieved by three-dimensional (3D) printing, can suppress conductive and convective heat transfer modes to a certain degree. However, formation of rigid structural nano-foams is of critical importance for the structure with further improved thermal insulation performance with acceptable mechanical strength. Benefitted from the Knudsen effect [1,13], thermal transport via gas medium can be greatly suppressed when the pore size is comparable to the mean free path of gas molecules, which is of the order of 100 nm. Therefore, in this study, we fabricate PLA based multiscale 3D freeform structures by combining 3D printing technology, and subsequent two-step gas foaming process to form nano-cellular foams within the 3D printed features towards the potential application for thermal insulation. The main focus of this research is to mainly evaluate the mechanical effect of nano-foams implemented within 3D printed structures as a first step.

## 2. Experimental Apparatus and Method

### 2.1. Freeform Structure

The freeform, an arbitrary 3D structure, is made of thin layers of microscale thickness, fabricated using a commercial 3D printer (3DKorea, Seongju, Korea). The used 3D printer can fabricate structures up to the dimension of 200 × 200 × 220 mm^3^ by stacking each layer with 25 microns thickness resolution. The filaments have a diameter of 1.75 mm, and various materials such as polylactic acid (PLA), acrylonitile butadiene styrene (ABS), polyether imide (PEI) and polyethylene terephthalate (PET) are compatible.

PLA, an eco-friendly and biodegradable resin based on natural raw material, was used in this study, and it is a favorable material for 3D printing with little toxicity. Low degree of material shrinkage and material residue at the nozzle, and homogeneous structure with suppressed bubble generation are additional merits. The PLA material was used as received from a 3D printer manufacturer (Rokit, Seoul, Korea), modified from the raw material supplied (NatureWorks, Minnetonka, MN, USA). The test samples were manufactured at a nozzle temperature of 230 °C, a bed temperature of 80 °C, a nozzle diameter of 0.4 mm, a printing speed of 70 mm/s, and a filament extrusion speed of 120 mm/s. To control the resolution of filament stacking, G-code was generated by adjusting the magnification with a three-dimensional printing open source code (Cura slicer program, Geldermalsen, The Netherlands). Designed space intervals between studs in freeform can be controlled with 3D computer-aided design (CAD) program such as SolidWorks and free CAD software 123D (Autodesk, San Rafael, CA, USA) for adjustment of mechanical and thermal properties (e.g., stiffness, strength, vibration damping, and thermal insulation). The properties of the freeform foam depend on structural geometry, resin composition, and inner cell (pore) size that can be controlled by foaming process parameters and type of filling gas. In this study, one type of simple 3D freeform structure was fabricated by stacking parallel lines in different angles by a 3D rapid prototyping (3D printing) technique to evaluate the effect of nano-foamed implemented. The dimension of fabricated 3D structure was 36.0 × 36.0 × 5.4 mm^3^.

### 2.2. Nanoscale Foaming Process

**Figure 1:**
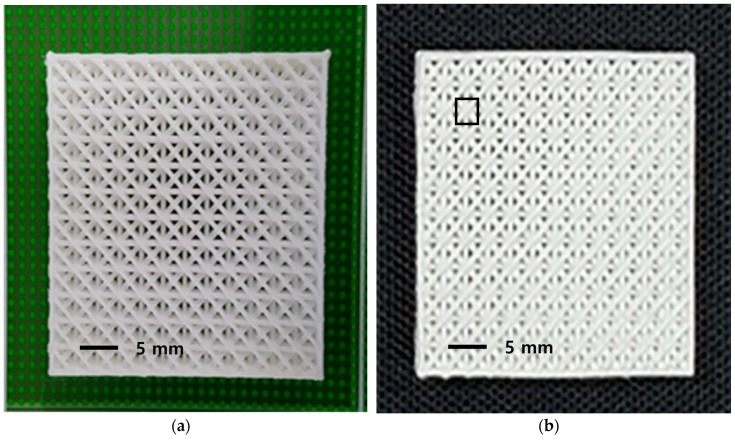
The solid freeform fabricated by 3D printing technology (**a**); and foamed structure after 3D printing via gas foaming process (**b**).

Nanoscale pores were foamed within the 3D printed freeform through the following procedure (detailed gas foaming procedures are found in the previous publication by authors [14]). First, liquid carbon dioxide maintained at 5 MPa and 20 °C was fed into a pressure vessel and compressed into 10 MPa. A high-pressure reaction chamber containing sample(s) was inserted into cold ethanol/water bath, maintained at temperature of −20 °C. High pressure was maintained during saturation process by continuous pumping with automatic pressure regulation. Saturation time of 24 h, a sufficiently long time based on the sorption kinetics, was applied. After the pressure vessel was depressurized quickly (at the estimated pressure drop rate of ~50–100 MPa/s), the sample was taken out from the vessel and immersed into a water bath set at designated foaming temperatures ($T_{f}$) for 3 min, followed by quenching in ethanol/water mixture of −20 °C for 1 min to freeze any further cell (pore) growth. The post-foaming temperature ($T_{f}$) was varied from 20 °C to 80 °C to seek its dependence on the foaming trend. Finally, the samples were wiped to remove residual ethanol/water mixture and were dried in an oven at 50 °C. An example freeform specimen before and after post-foaming process can be seen in Figure 1.

### 2.3. Measurement Apparatus and Method 

#### 2.3.1. Microstructure Observation

To understand the cross-sectional structure of the foams implemented within 3D printed freeform structure, the specimens were cut at low temperature using liquid nitrogen, and the cut surface was sputter-coated with platinum of 15 nm thickness. Then, the specimens were observed by a field emission scanning electron microscope (FE-SEM, SUPRA 55VP model, Oberkochen, Germany) with typical acceleration voltage of 2.0 kV.

#### 2.3.2. Thermal and Pyrolysis Measurements

To conduct the thermal analysis at as-printed and as-foamed states of the specimen, one cycle test was performed using differential scanning calorimetry (Model Discovery, TA Instruments, New Castle, DE, USA) based on the ASTM standard (D3417-83, D3418-82). To investigate the weight ratio of organic components contained and the thermal stability at high temperature, the degree of thermal decomposition was measured with a SDT analyzer (simultaneous differential scanning calorimeter and thermogravimetric analysis, Universal V4.5A model, TA Instruments, New Castle, DE, USA). The heating rate was set at 10 °C/min and the weight and energy difference of the specimens were measured for the temperature range of 30–600 °C under nitrogen environment.

#### 2.3.3. Measurement of Mechanical Strength

Bending and compressive tests were carried out to evaluate the mechanical properties of the fabricated structures. For the mechanical strength measurement, a universal material testing machine (UTM, LR 50K model, LLOYD Instruments, West Sussex, UK) was used, and at least four specimens were prepared for each experimental group. In each test, the crosshead speed was set based on the ASTM standard (ASTM D6272, D695).

## 3. Results and Discussion

### 3.1. Microstructure of Freeform Foam

**Figure 2:**
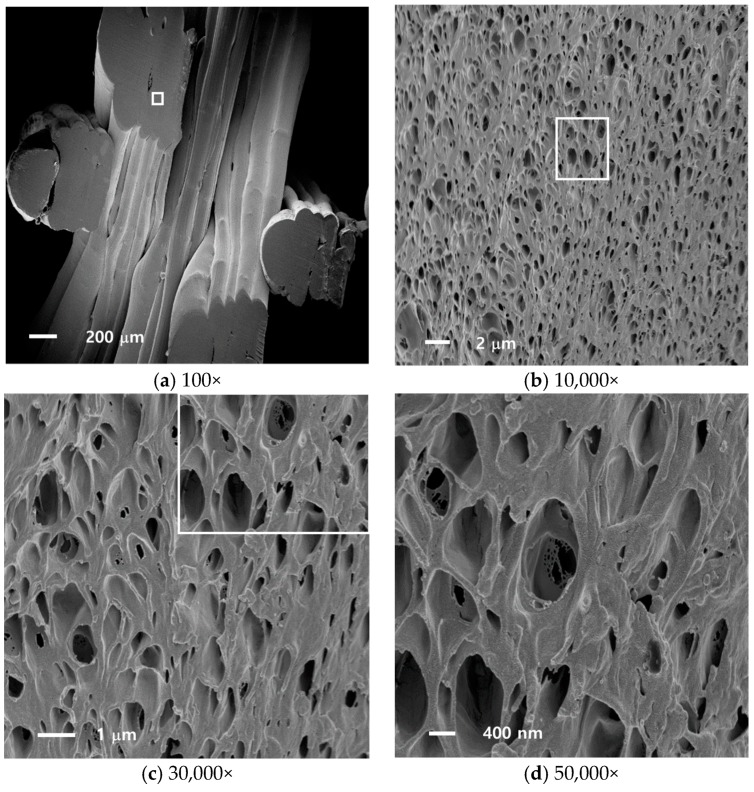
SEM micrographs of a 3D printed and foamed PLA structure (saturation: −20 °C, post-foaming: 60 °C): box in (**a**) denotes zoomed-in area in (**b**); box in (**b**) denotes zoomed-in area in (**c**); and box in (**c**) denotes zoomed-in area in (**d**).

Figure 2 shows cross-sectional FE-SEM images of a fabricated 3D structure with nano-foams at the enlargement of 100×, 10,000×, 30,000× and 50,000×. It is observed that closed cells (pores) of various sizes are randomly distributed within the specimen, and a relatively small amount of polymer matrix is surrounding the hollow pores (cells), forming independent pores separated by robust interfaces. It is seen that carbon dioxide formed small pores in high density, and the pores can be grown to relatively larger sized pores through nucleation and cell growth processes. The structural flexibility of specimens is attributed to nanoscale pores, as visualized in Figure 2b–d.

**Figure 3:**
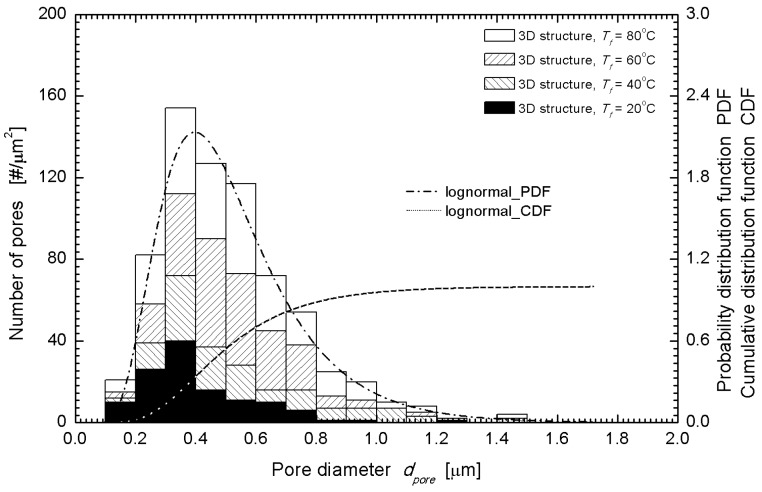
Size distribution of pores and probability density function for a 3D printed and gas foamed PLA structure (saturation: −20 °C, post-foaming: 20–80 °C), measured from SEM cross-section images (e.g., Figure 2).

To obtain further information on the pore structure and pore density from the SEM images, the ImageJ and MorphoLibJ package (NIH, Bethesda, MD, USA) libraries were used. The measured cross-sectional areas were assumed to be circular, and the effective diameters were calculated using the calculation formula, $d_{pore}$= $\sqrt{4A/\pi }$, where *A* is the cross-sectional area, and $d_{pore}$ denotes the effective pore(cell) diameter. The pore size distribution obtained in this manner was fit to logarithmic normal distribution as many natural phenomena are well fit [14], and the number average diameter was obtained. To estimate the number density of the pore formation (closed cell), the number of pores in a 100 μm^2^ area was counted, by including at least 100 pores (interrogation areas were selected from various regions from center to edge to find representative characteristics). In the case of PLA, the size of the pore showed minor variations, similar to the case of ABS, but the probability density distribution function exhibited larger difference [14]. The overall lognormal distribution fitting parameters were the mean diameter of 523.7 nm and variance of 0.05546 as shown in Figure 3.

**Figure 4:**
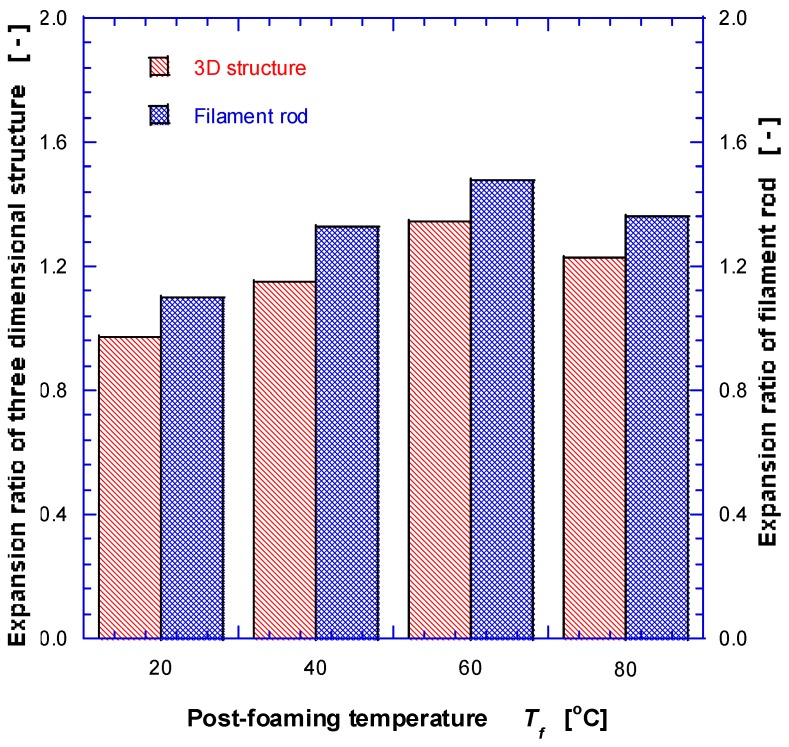
Measured expansion ratios for fabricated 3D structures and free rod at various post-foaming temperatures of PLA ($P_{s}\;$= 10 MPa, $T_{s}\;$= −20 °C).

The sample was saturated at −20 °C and 10 MPa for 24 h, then suddenly depressurized (at the rate of ~50–100 MPa/s), and finally the foaming process was carried out for 3 min at 20 °C, 40 °C, 60 °C and 80 °C, respectively. Quenching in the thermostat-controlled bath at −20 °C was followed to suppress further foaming, and finally dried in a convection oven set at 50 °C. The resulting sizes of the three-dimensional structures and filaments were measured, and the calculated expansion ratios are plotted in Figure 4. The volume expansion ratio $e_{r}$, based on the mass and apparent volume in the case of the 3D grid structure (and radial expansion ratio of the rod in the filament rod), has been calculated using the following formula.

(1) $$e_{r}\;=\;\frac{V_{f}}{V_{p}}≅\;\frac{\rho_{p}}{\rho_{f}}\;=\;\frac{1}{\rho_{r}}$$

At the foaming temperature of 20 °C, the expansion ratio was very limited, but increased at higher temperature up to 60 °C. However, at 80 °C, it reduced vs. at 60 °C due to the glass transition at ~60 °C for the PLA; part of expanded pores was coalesced and/or ruptured, and part of open cell structures was shrunk and rearranged. It has been found that the swelling ratio of 3D structures totally constrained to each other is lower than that of the filament rod free from external stress. In addition, three-dimensional structures were expanded to the out-of-plane direction as well because of stacked flat shape of extruded polymer and internal residual stress. It has also been found that the swelling ratio of the cylindrical rod increases by ~7–14% compared with that of 3D structure, as shown in Figure 4.

**Figure 5:**
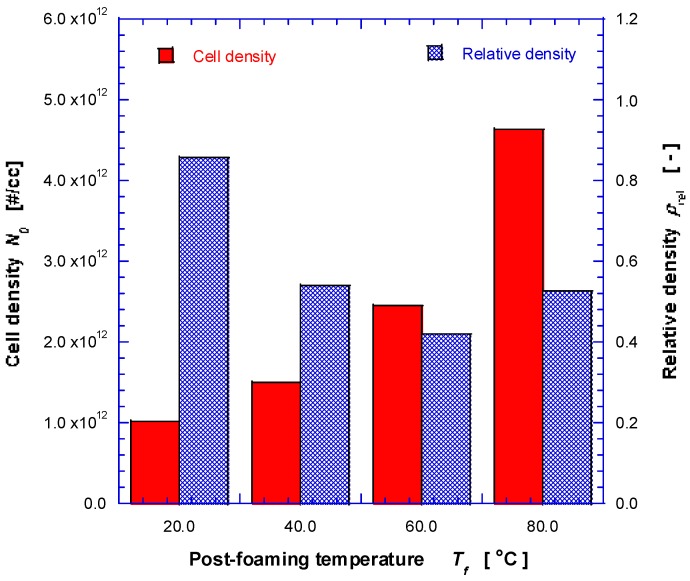
Effect of post-foaming temperature on cell (pore) density and relative density measured from SEM cross-section images (e.g., Figure 2).

The cell (pore) density $N_{0}$, i.e., the number of cells (pores) per unit volume of the foam, and relative density (i.e., *solid fraction* = 1 − *void fraction*), can be estimated from the following correlations:

(2) $$N_{0}=\;{\left(\frac{nM^{2}}{A}\right)}^{3/2\;}\;\times \;\left(\frac{1}{1-V_{f}}\right)\;=\;{\left(\frac{nM^{2}}{A}\right)}^{3/2}\times \;(\frac{\rho_{p}}{\rho_{f}}\;),$$

(3) $$V_{f}\;=\;1-\left(\frac{\rho_{f}}{\rho_{p}}\;\right)\;=\;1-\rho_{r},$$

where *n* is the number of cells (pores) in the probe volume, *A* is the probe area, *M* is the magnification, $V_{f}$ is the void fraction of foam, $\rho_{p}$ is the polymer density, $\rho_{f}$ is the foam density, and $\rho_{r}$ is the relative density. Foam densities of rods were measured by weight displacement method [14]; the samples were weighed on an analytical scale and immersed in water, and then volume of water displaced by the sample was then measured. For 3D structures, it was calculated from mass and apparent volume. As shown in Figure 5, the estimated cell (pore) density generally increases monotonically as $T_{f}$ increases, and the relative density decreases monotonically except for the sample prepared by post-foaming temperature of 80 °C. The relative density of sample prepared by $T_{f}$ = 80 °C is higher because fraction of pores has been coalesced, burst and shrunk considerably.

The reported true density of the polylactic acid mixed resin (Nature Works PLA) used for this study is ~1.24 g/cm^3^. Based on the dimension of 36.0 × 36.0 × 5.4 mm^3^ and a measured mass of 2.183 g for non-foamed freeform specimen, the bulk density is estimated at ~0.304 g/cm^3^. Thus, solid fraction of the sample fabricated by 3D printing without foaming step was ~24.5%, i.e., 0.304 (g/cm^3^)/1.24 (g/cm^3^). One can obtain approximate values on the solid fraction of 3D structures after foaming by multiplying the solid fraction (~24.5%) to the relative density shown in Figure 5 (solid fraction of foamed rib). Then, the void fraction, an important parameter for thermal and mechanical properties, can be estimated by subtracting from 100%.

### 3.2. Thermal and Pyrolysis Characteristics

**Figure 6:**
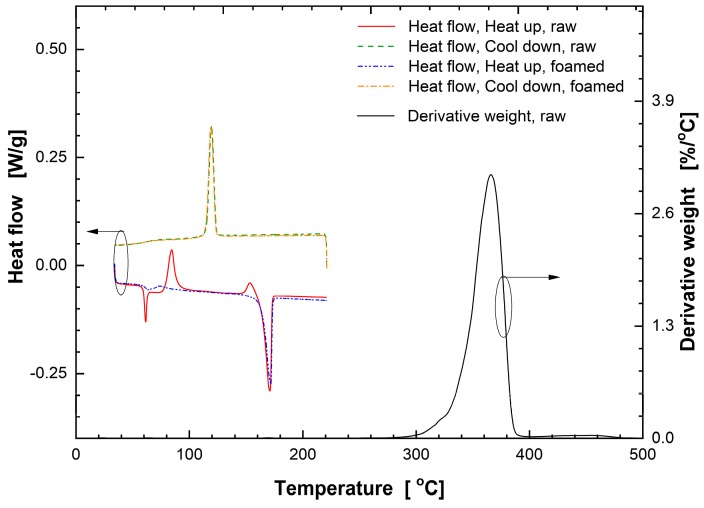
Thermogravimetric analysis (TGA) and digital scanning calorimetry (DSC) thermograms of the 3D printed freeform PLA specimens. Both non-foamed and foamed samples were tested.

The thermal analysis was performed for the 3D printed samples and subsequently foamed samples as shown in the left part (lower temperature range up to 220 °C) of Figure 6. One cycle test was conducted to investigate the precise state of PLA, i.e., for experimental measurement of glass transition temperatures before and after gas foaming process, using differential scanning calorimetry (Model Discovery, TA Instruments, New Castle, DE, USA). The analysis was carried out in an aluminum container in a nitrogen atmosphere based on the regulations in ASTM D 3417-83 and D 3418-82. The sample weight of 2–3 mg was used. Samples were heated from 34 °C up to 220 °C at the ramping rate of 2 °C/min, held at 220 °C for 5 min, and cooled down to 34 °C at the rate of 2 °C/min, completing one cycle.

During heating of the non-foamed specimen, as can be seen in Figure 6, the initial phase transition takes place at 56.4 °C, which corresponds to the glass transition temperature in which the polymer transits to a highly elastic state. The endothermic peak at 170.9 °C indicates melting process. Observation of thermal transitions at temperature peaks of 84.4 °C and 153.7 °C are associated with cold crystallization. Cold crystallization is caused by the rearrangement of molecular chains in the crystalline PLA layer assisted by increased mobility during the manufacturing and heating processes. Since the polymer is in an amorphous state after the heating stage, this phase transition is present. During the cooling process, the phase transition occurs at 119.3 °C. It is presumed that the fabricated rigid freeform after extrusion from the nozzle and cooling is in amorphous and partially crystalline states. Moreover, the observed amorphous state is independent of the cooling rate because the heating and cooling speed of the DSC is as slow as 2 °C/min.

On the other hand, the foamed specimens subjected to the saturation and post-forming processes show a clear difference in that carbon dioxide was contained or plasticization occurred during the post-foaming process so that no cold crystallization occurs due to rearrangement of molecular chains during the heating process.

Pyrolysis measurements were performed using a SDT (simultaneous DSC/TGA, TA Instruments, New Castle, DE, USA) analyzer, as also shown in Figure 6. The maximum endothermic reaction occurring at 371.78 °C is assigned to the mixed components during evaporation and production of filament for 3D printing. As observed in the pyrolysis curve obtained by thermogravimetric analysis (TGA), the PLA, a constituent of freeform foam, starts to evaporate at ~297.3 °C (weight ratio of 99 wt.%), and weight loss rate is maximized at ~366 °C (~3.05 wt.%/°C), reaching almost full evaporation at ~431.5 °C (weight ratio of 1 wt.%) ultimately reaching negligible weight loss rate. Major pyrolysis event takes place between 338.6 and 376.5 °C, where ~80% of total mass is lost and most latent heat of evaporation is consumed. It is noted that, for temperature higher than 277.1 °C, thermal decomposition rate of filament material rapidly increases (>0.01 wt.%/°C), resulting in a large mass loss, thus setting the upper limit of working temperature for 3D printed features based on PLA.

On the other hand, morphology of PLA crystal is influenced by composition and thermal history. PLA crystals grown at different temperatures go through regime changes associated with nucleation and crystal growth rates [15]. While the slow quiescent crystallization kinetics of PLA may limit its processing speed for some applications, utilizing stress-induced crystallization (SIC) with PLA has been success commercially because of naturally wide processing window. It is possible to achieve highly oriented PLA in the rubbery or molten state during 3D printing operations such as fiber extrusion, layer bonding, and/or shear scrubbing. Such fabrication routes take full advantage of the semi-crystalline nature of PLA to develop different properties. To estimate a degree of crystallinity, the following equation is used:

*DegreeofCrystallinity* = (*∆H_m_−∆H_c_*)/*∆H_m,max_,* (4)

where *∆H_m_* is measured heat of fusion and *∆H_c_* is heat of crystallization. Most commonly, an enthalpy of fusion 93.1 J/g is used for a 100% crystalline poly-l-lactide (PLLA) or poly-d-lactide (PDLA) homo-polymers having infinite crystal thickness throughout the PLA literatures (i.e., *∆H_m,max_* = 93.1 J/g was used here). From digital scanning calorimetry (DSC) scans, percent crystallinity of 26.88% is estimated for the fabricated freeform specimen.

### 3.3. Mechanical Properties

**Figure 7:**
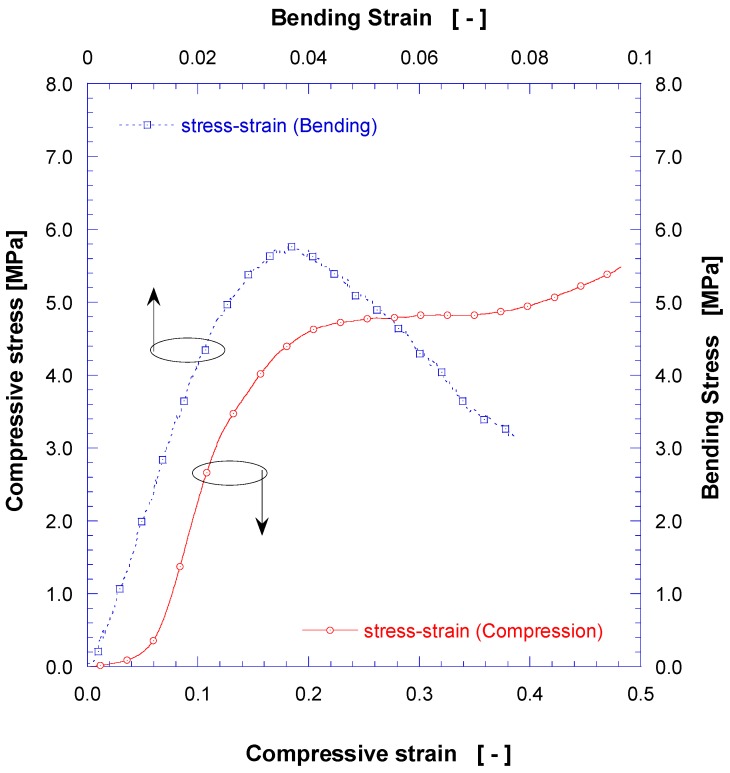
Bending and compressive stress-strain curves for PLA solid freeform before foaming process.

To understand the mechanical properties of 3D structures fabricated by 3D printing, representative compressive and bending test results are displayed in Figure 7 (note difference in *x*-axis scales). In the result of four-point bending test, the bending stress of freeform specimen showed an elastic behavior up to a bending strain of ~0.025 mm/mm with an initial tangential elastic modulus of 204.3 MPa. The maximum bending stress reached ~5.65 MPa at ~0.035 mm/mm, and then gradually decreased. The maximum bending strain at which fracture occurred was ~0.07 mm/mm. The main reasons for improved mechanical strength vs. typical insulating foams are: (i) the 3D printed structure of polylactic acid polymer has a relatively strong bonding force between molten layers; and (ii) the flexible freeform structure, as staggered grid studs, withstands a large strain. The dimension of specimen before compression test was 36.0 × 36.0 × 5.4 mm^3^. The maximum compressive deflection was limited to ~4.2 mm, which was a machine limit for the protection of measuring equipment with overload protection during compression. In compressive test, freeform specimens showed linear elastic behavior for the compressive strain range of ~0.07–0.12 mm/mm with an initial tangential elastic modulus of 52.67 MPa (coefficient of determination in linear modulus *R*^2^ = 0.9986), and flat region for the strain range of 0.22–0.38 mm/mm with constant compressive stress value of ~4.81 MPa. The specific compressive strength and specific compressive modulus of elasticity were calculated as 15.82 MPa · cc/g and 169.67 MPa · cc/g respectively. In the compression test, the specimen did not rupture rapidly until strain reached about 25% because the space between inner grids in polymer matrix studs can accommodate and withstand high strains maintaining the connectivity.

**Figure 8:**
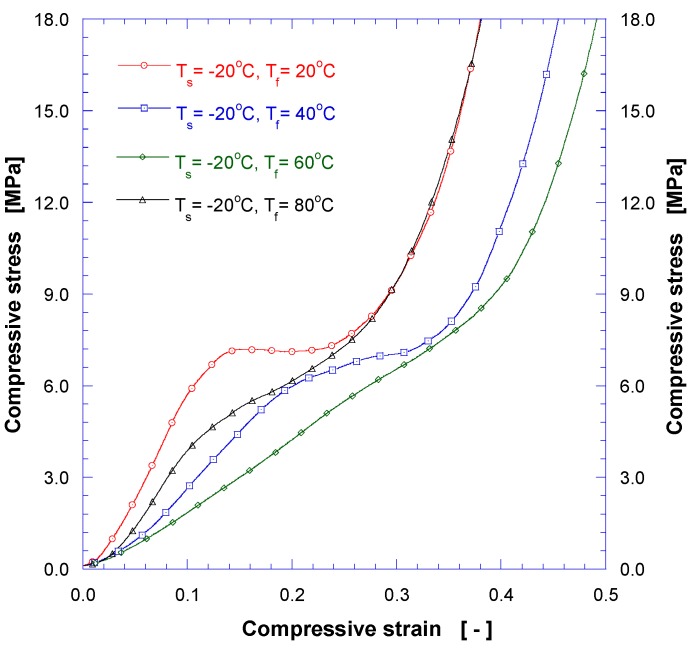
Compressive stress-strain curves for solid freeform PLA after foaming process.

**Figure 9:**
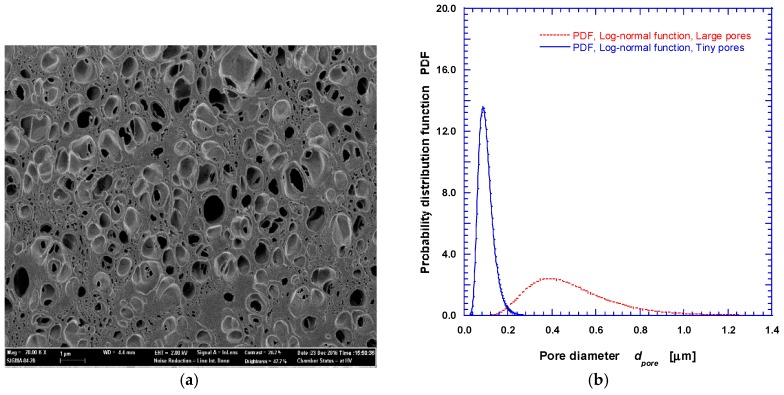
SEM image (**a**); and size distribution (**b**) of multiscale pores in nano-cellular PLA rib prepared at $T_{s}$ = −20 °C and $T_{f}$ = 80 °C.

In post-foaming process, 3D printed structures may experience nonuniform deformations due to internal residual stress and/or nonuniform temperature distribution within the structure during the foaming process. We have used a jig of fine mesh and/or grill with a very flexible spring during the foaming process to partly resolve the deformation issues. The measured compressive strength of the PLA foam specimen is shown in Figure 8. It is observed that the modulus of elasticity decreases as the post-foaming temperature increases up to 60 °C, and increases at $T_{f}$ = 80 °C. The highest mechanical strength of CO_2_ saturated grid sample is greater than that of pristine one presumably because crystals are rearranged due to the plasticization effects by CO_2_ impregnation. The maximum compressive stress increased by about 50%, from 4.8 MPa to 7.2 MPa. As previously described, when the post-foaming temperature exceeds glass transition temperature ($T_{g}$) of PLA, the cell (pore) is partially expanded and ruptured leading to reduced foam size. As shown in an SEM image (Figure 9a) for the post-foaming temperature of 80 °C, large pores were formed in an irregular fashion, and some of them were burst, forming open cells. The SEM image in Figure 9a was analyzed to obtain the pore size distribution shown in Figure 9b. It forms dual diameter distributions with mean diameters of 490.8 nm and 100.5 nm, confirming the formation of multi-scale structure and the possibility of controlling physical properties by suitable tuning of foaming conditions. Further research on the controlling mechanisms of the cell size and closed/open cell ratios is under way.

## 4. Conclusions

In this study, a new type of PLA based 3D structures has been fabricated by integrating 3D printing and gas foaming technologies. Through morphological characterization, formation of multiscale porous structure with nanoscale cellular pores has been confirmed, as a highly beneficial aspect towards thermal insulation with lightweight and reasonable mechanical strength. The highest mechanical strength of CO_2_ saturated grid sample was greater by ~50% compared with that of pristine one possibly due to rearrangement of crystals. Through variation of post-foaming temperature, it was found that the post-foaming temperature higher than the glass transition temperature deteriorate mechanical strength, providing a process guideline. Thermal decomposition of filament material set the upper limit of temperature for 3D printed features based on PLA, characterized by simultaneous DSC/TGA analyzer. Potential to control and tune porosity of the nano-foams within 3D printed PLA structure has been examined. Further efforts to measure thermal insulation performance of the fabricated structures are under way.
